# Supplementary material for: Expression of foetal gene Pontin is essential in protecting heart against pathological remodelling and cardiomyopathy
Source: Nat Commun. 2025 Feb 14;16:1650. doi: 10.1038/s41467-025-56531-4 (PMC11829043; doi:10.1038/s41467-025-56531-4)
Supplement: Supplementary file 1 — Supplementary Information [file 41467_2025_56531_MOESM1_ESM.pdf]

SUPPLEMENTARY INFORMATION

Expression of foetal gene Pontin is essential in protecting heart against pathological remodelling and cardiomyopathy

Bayu Lestari[1,#], Ardiansah Bayu Nugroho[1,#], Thuy Anh Bui[1,#], Binh Nguyen[1], Nicholas Stafford[1,2], Sukhpal Prehar[1], Min Zi[1], Ryan Potter[1], Efta Triastuti[1], Florence M. Baudoin[1], Alicia D'Souza[3], Xin Wang[1], Elizabeth J. Cartwright[1], Delvac Oceandy[1,*].

[1]Division of Cardiovascular Sciences, Faculty of Biology, Medicine and Health, The University of Manchester, Manchester Academic Health Science Centre, Manchester, United Kingdom

[2]Division of Diabetes, Endocrinology and Gastroenterology, Faculty of Biology, Medicine and Health, The University of Manchester, Manchester Academic Health Science Centre, Manchester, United Kingdom

[3]National Heart and Lung Institute, Imperial College, London, United Kingdom

[#]These authors contributed equally to this work

Supplementary tables 1 – 4

Supplementary figures 1 – 13

Supplementary Table 1 Clinical information of human heart tissue samples

Group	No	Sex	Clinical Diagnosis	Heart tissue diagnosis
Heart failure	1	Male	Congestive heart failure	Hypertrophy
	2	Male	Congestive heart failure	Hypertrophy, interstitial fibrosis, chronic ischaemic changes
	3	Male	Congestive heart failure	Hypertrophy, interstitial fibrosis
Non-heart failure	1	Male	Adenocarcinoma colon	Normal
	2	Male	Head injury	Normal
	3	Male	Metastatic lung cancer	Normal

Supplementary Table 2 List of antibodies used in this study

Antibodies	Species	Source	Dilution
**Western blot**			
RUVBL1	Rabbit polyclonal	Proteintech (10210-2-AP)	1:1000
MST1	Rabbit monoclonal	Cell Signaling Technology (#3682)	1:1000
Phospho-MST1 (Thr183)/MST2 (Thr180)	Rabbit polyclonal	Cell Signaling Technology (#49332)	1:1000
LATS1	Rabbit polyclonal	Proteintech (#17049-1-AP)	1:1000
Phospho-LATS1 (Ser909)	Rabbit monoclonal	Cell Signaling Technology (#9157)	1:1000
SAV1	Rabbit monoclonal	Cell Signaling Technology (#13301)	1:1000
MOB1	Rabbit monoclonal	Cell Signaling Technology (#13730)	1:1000
Phospho-MOB1 (Thr35)	Rabbit monoclonal	Cell Signaling Technology (#8699)	1:1000
Non-phospho (Active) YAP (Ser127)	Rabbit monoclonal	Cell Signaling Technology (#29495)	1:1000
Phospho-YAP (Ser127)	Rabbit monoclonal	Cell Signaling Technology (#4911)	1:1000
YAP antibody	Mouse monoclonal	Santa Cruz Biotechnology (#sc-376830)	1:1000
P53	Mouse monoclonal	Santa Cruz Biotechnology (#sc-47698)	1:1000
Bax	Mouse monoclonal	Santa Cruz Biotechnology (#sc-20067)	1:1000
Bad	Rabbit monoclonal	Cell Signaling Technology (#9292)	1:1000
Bcl-xL	Rabbit monoclonal	Cell Signaling Technology (#2762)	1:1000
Caspase-3	Rabbit monoclonal	Cell Signaling Technology (#9662)	1:1000
GAPDH (HRP-linked)	Rabbit	Cell Signaling Technology (#3683)	1:5000
β-actin (HRP-linked)	Rabbit	Cell Signaling Technology (#5125)	1:5000
Vinculin	Rabbit	Abcam (#ab129002)	1:1000
Anti mouse IgG (HRP-linked)	Mouse	Cell Signaling Technology (#7076)	1:5000
Anti rabbit IgG (HRP-linked)	Rabbit	Cell Signaling Technology (#7074)	1:5000
**Immunofluorescence**			
Ki67	Rabbit	Abcam (#Ab15580)	1:100
Sarcomeric α-actinin	Mouse monoclonal	Sigma-Aldrich (#A7811)	1:100
pH-H3	Rabbit polyclonal	Invitrogen (#PA5-17869)	1:100
Alexa Fluor 647 anti-mouse	Mouse polyclonal	Jackson Immuno Research (#115-605-072)	1:200
Alexa Fluor 488 anti-rabbit	Rabbit polyclonal	Jackson Immuno Research (#711-545-152)	1:200

Supplementary Table 3 List of primers used in qPCR analysis

Genes	Species	Forward primer (5' – 3')	Reverse primer (5' – 3')
Ruvbl1	Human	AGGTGAAGAGCACTACGAAGA	CTACTATGACGCCACATGCCT
GAPDH	Human	GGAGCGAGATCCCTCCAAAAT	GGCTGTTGTCATACTTCTCATGG
LATS1	Mm	AAAGCCAGAAGGGTACAGACA	CCTCAGGGATTCTCGGATCTC
LATS2	Mm	GGACCCCAGGAATGAGCAG	CCCTCGTAGTTTGCACCACC
MST1	Mm	TCATTCGGCTACGGAACAAGA	GACCTGCGACTCCAAAGTCTG
MST2	Mm	CGGGGTCCGTTTCAGACATAA	GCGTTTTGCCATTGTATCTGTT
YAP1	Mm	ACCCTCGTTTTGCCATGAAC	TGTGCTGGGATTGATATTCCGTA
ANP	Mm	GCTTCCAGGCCATATTGGAG	GGGGGCATGACCTCATCTT
Col-1α	Mm	GCTCCTCTTAGGGGCCACT	CCACGTCTCACCATTGGGG
Bax	Mm	TGAAGACAGGGGCCTTTTTG	AATTCGCCGGAGACACTCG
Bad	Mm	AAGTCCGATCCCGGAATCC	GCTCACTCGGCTCAAACTCT
Caspase-3	Mm	ATGGAGAACAACAAAACCTCAGT	TTGCTCCCATGTATGGTCTTTAC
p53	Mm	CTCTCCCCCGCAAAAGAAAAA	CGGAACATCTCGAAGCGTTTA
Bcl-xL	Mm	GACAAGGAGATGGCAGGTATTGG	TCCCGTAGAGATCCACAAAAGT
Bcl-2	Mm	ATGCCTTTGTGGAACTATATGGC	GGTATGCACCCAGAGTGATGC
Ankrd1	Mm	GCTGGTAACAGGCAAAAAGAAC	CCTCTCGCAGTTTCTCGCT
	Rat	GCTGGTAACGGGCAAAAAGAAC	CCTCTCGAACTTTCTCACT
Birc5	Mm	GAGGCTGGCTTCATCCACTG	CTTTTTGCTTGTTGTTGGTCTCC
Cyr61	Mm	CTGCGCTAAACAACTCAACGA	GCAGATCCCTTTCAGAGCGG
Fgf2	Mm	GCGACCCACACGTCAAACTA	TCCCTTGATAGACACAACTCCTC
Ctgf	Rat	GGGCCTCTTCTGCGATTTC	ATCCAGGCAAGTGCATTGGTA
Pikcb3	Mm	CTATGGCAGACAACCTTGACAT	CTTCCCGAGGTACTTCCAACT
	Rat	CTATGGCAGACACCCTTGACAT	CTTCCCGGGGTACTTCCAACT
Tead1	Mm	AAGCTGAAGGTAACAAGCATGG	GCTGACGTAGGCTCAAACCC
	Rat	AAGCTAAAGGTAACAAGCATGG	GCTGATGCAGGCTCAAACCC
GAPDH	Mm	AGGTCGGTGTGAACGGATTTG	TGTAGACCATGTAGTTGAGGTCA

Supplementary Table 4 Primer sequences and size of PCR products for the identification of Pontin$^{flox/flox}$, αMHC-MerCreMer, and Pontin^{icKO} mice

Primer	Sequence (5'-3')	Amplicon size (bp)		
		Wild-type	Transgenic	Knock-out
**Cre WT**				
Forward	TCTATTGCACACAGCAATCCA	295	-	-
Reverse	CCAACTCTTGTGAGAGGAGCA			
**Cre Transgene**				
Forward	TCTATTGCACACAGCAATCCA			
Reverse	CCAGCATTGTGAGAACAAGG	-	300	-
**Pontin flox/flox**				
Forward (Ef-3992)	CCTTCCCTGAGTGCTGATTTCT	240 (WT)	-	-
Reverse (Er-3995)	GGGGTAATACACAGCCCAAGAG	414 (L2)		
**Pontin cKO**				
Forward (Ef-3990)	TTCCACAGCTCTCGCCTGGGCA	1071 (WT)		
Reverse (L3r-3991)	CCACATATGCTCTACTGCTGAA	1276 (L2)	-	458

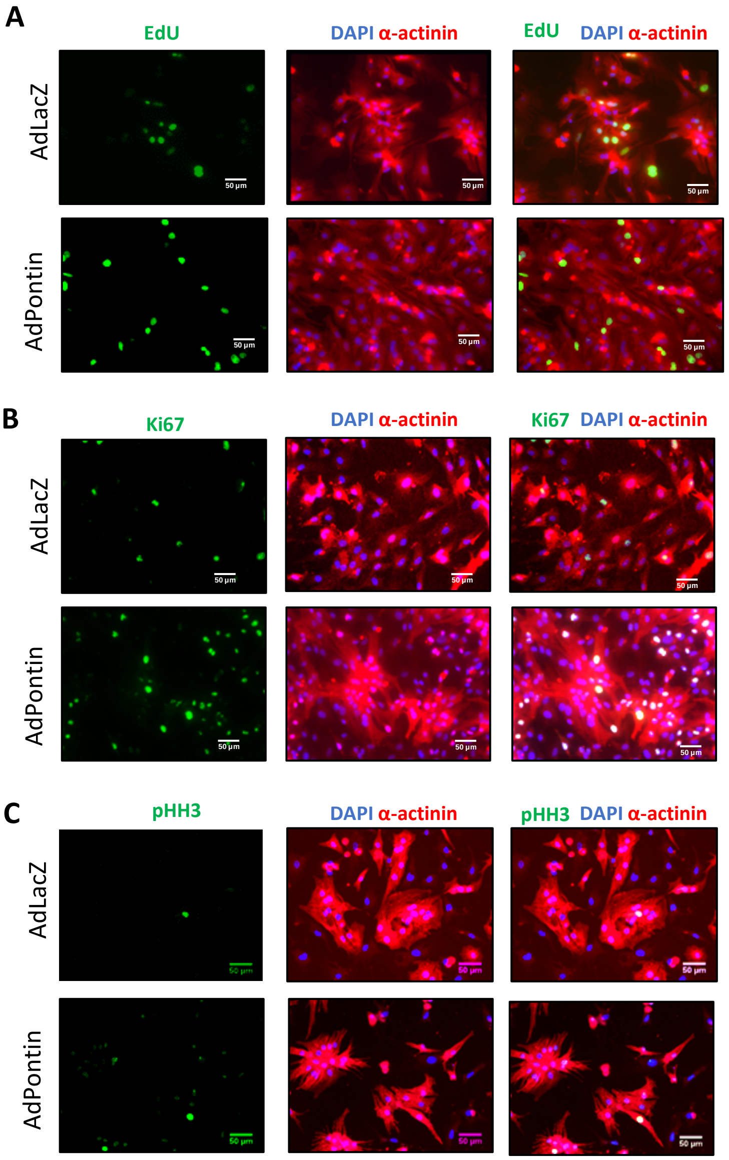

Supplementary figure 1. Split channel fluorescence images on the effects of Pontin overexpression on cell proliferation. A) EdU staining (green channel), DAPI and α-actinin stainings (blue and red) and merge image showing the effects of Pontin overexpression on EdU incorporation. **B)** Representative images of Ki67 (green channel) with corresponding DAPI + α-actinin stainings (blue and red) and merge image. **C)** Staining of cardiomyocytes with pHH3 (green channel) DAPI and α-actinin stainings (blue and red staining) and merge image indicating an increase in cell proliferation following Pontin overexpression. (scale bars=50μm)

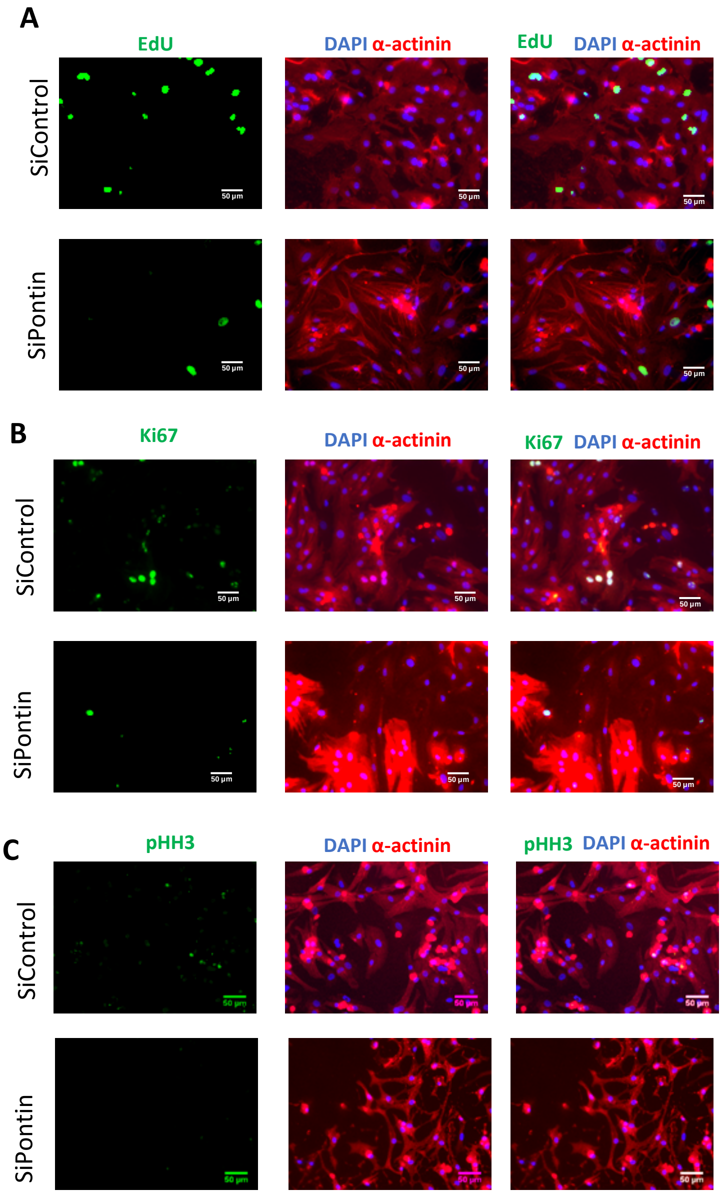

Supplementary figure 2. Split channel fluorescence images showing the effect of Pontin gene silencing on cell proliferation. A) Separate channel images of EdU staining (green channel), DAPI and α-actinin stainings (blue and red) and merge image on NRCM after treatment with siPontin or siControl. **B)** Split channel images displaying Ki67 staining (green channel), DAPI + α-actinin stainings (blue and red) and merge image. **C)** Example images of pHH3 staining (green channel) DAPI and α-actinin stainings (blue and red) and merge image in cardiomyocytes lacking Pontin. (scale bars=50μm)

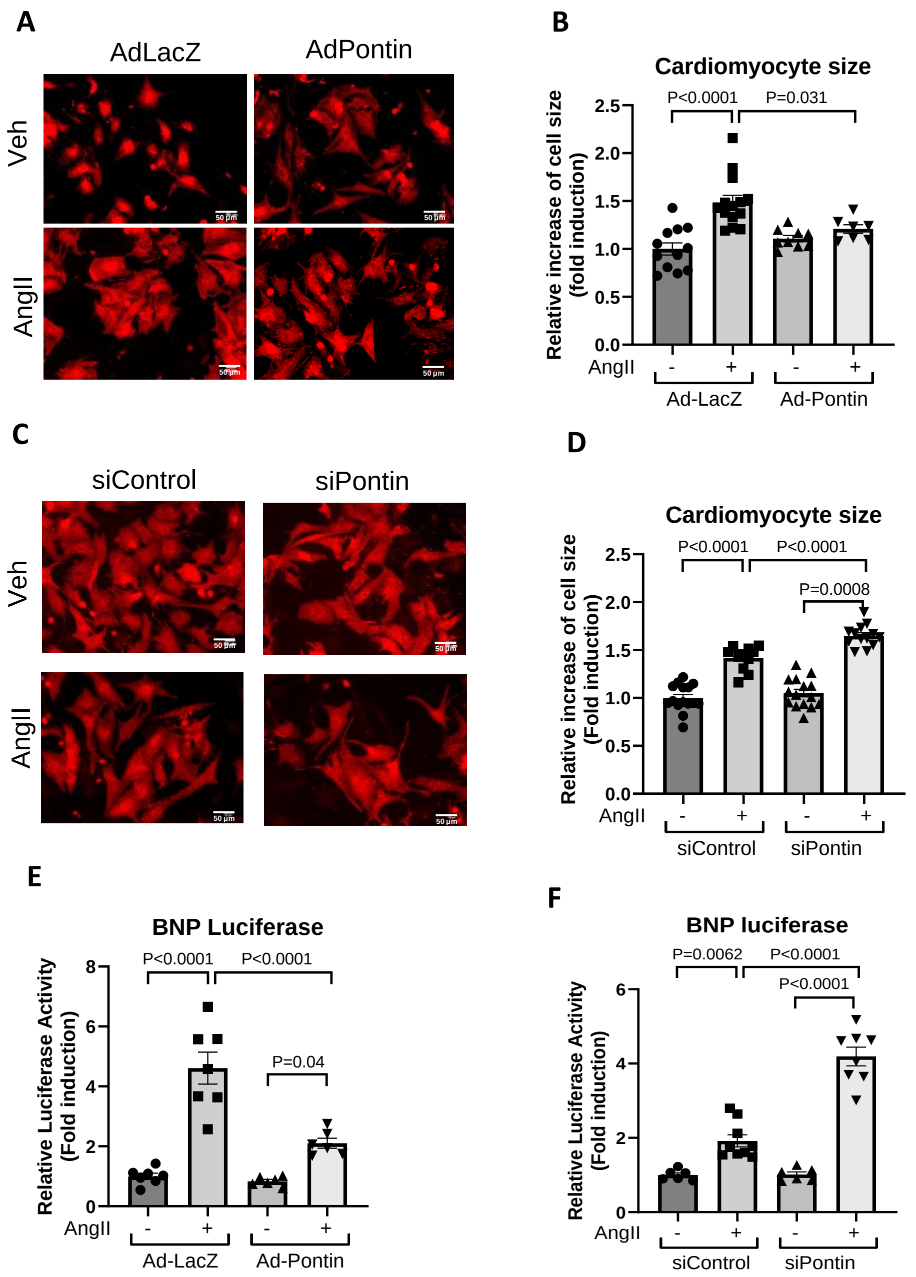

Supplementary figure 3. Pontin regulates Angiotensin II induced cardiomyocyte hypertrophy. **A)** Example images of NRCMs folowing stimulation with Ang II (1µM, 48h). Cells were stained with anti-αactinin antibody. Pontin overexpression was achieved using adenovirus overexpression system (AdPontin) whereas adenovirus expressing LacZ (AdLacZ) was used as control (scale bars=50µm). **B**) Quantification of cardiomyocyte size indicated that Pontin overexpression reduced hypertrophic response (LacZ Vehicle,n=12; LacZ AngII, n=14; Pontin Vehicle, n=9; Pontin AngII, n=7 samples). **C)** Representative images showing NRCMs treated with siRNA Pontin or siControl before stimulation with AngII (1µM, 48h). Cells were stained with anti-αactinin antibody (scale bars=50µm). **D)** Measurement of cell surface area indicated that Pontin gene silencing led to the exaggerated hypertrophic response (siControl Vehicle,n=15; siControl AngII, n=11; siPontin Vehicle, n=15; siPontin AngII, n=13 samples). **E**) Analysis using BNP luciferase reporter confirmed that Pontin expression reduced hypertrophic response (LacZ Vehicle,n=7; LacZ AngII, n=7; Pontin Vehicle, n=6; Pontin AngII, n=6 independent experiments), whereas **F)** Pontin knockdown increased hypertrophy (siControl Vehicle,n=6; siControl AngII, n=9; siPontin Vehicle, n=6; siPontin AngII, n=8 independent experiments). Data are presented as mean ± SEM. Statistical test used: one way ANOVA followed by multiple comparisons. Source data are provided as a Source Data file.

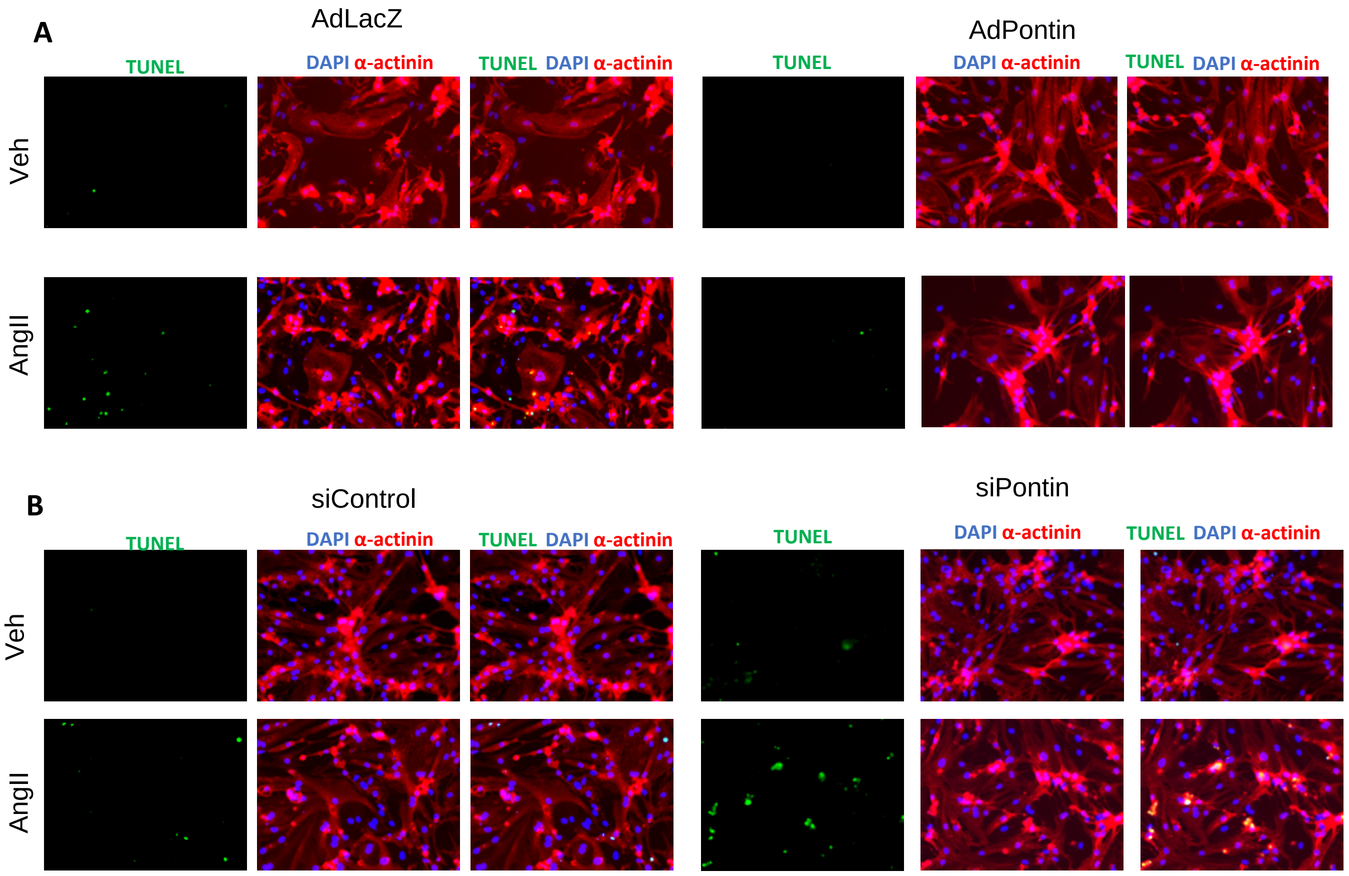

Supplementary figure 4. Split channel fluorescence images of TUNEL staining. A) Images of TUNEL staining (green channel), DAPI and α-actinin stainings (blue and red) and merge picture showing reduction of apoptosis in NRCM overexpressing Pontin (**A**) and in NRCM lacking Pontin (**B**).

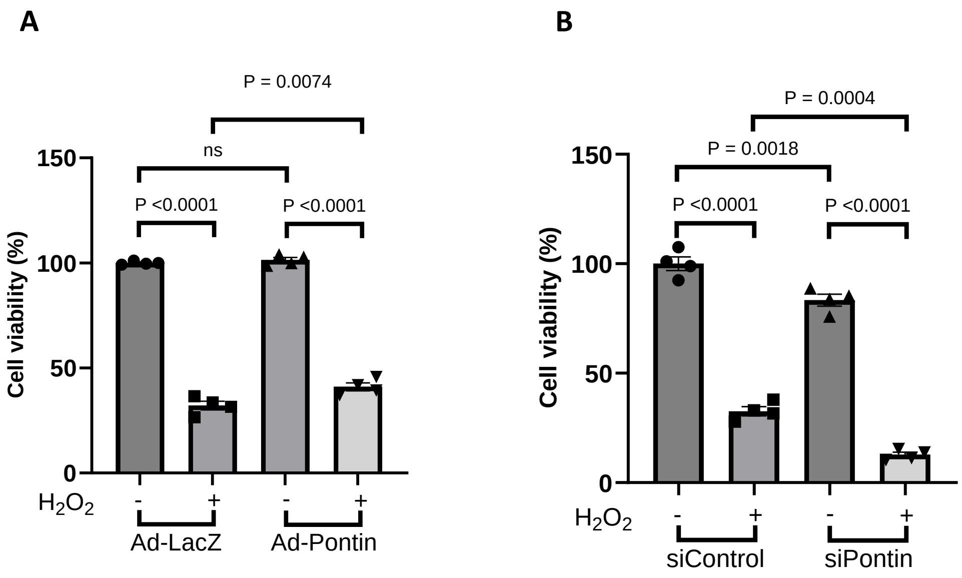

Supplementary figure 5. Pontin expression enhances cell viability following oxidative stres. A) Alamar blue assay was performed to assess cell viability following oxidative stress. NRCMs were treated with H_2O_2 (200 µM, 4 hours). Pontin overexpression (**A**) improved cell survival (n=4 independent experiments in each group) whereas Pontin gene knockdown (**B**) led to the reduction of cell survival following H_2O_2 treatment (n=4 independent experiments in each group). Data are presented as mean ± SEM. Statistical test used: one way ANOVA followed by multiple comparisons. Source data are provided as a Source Data file.

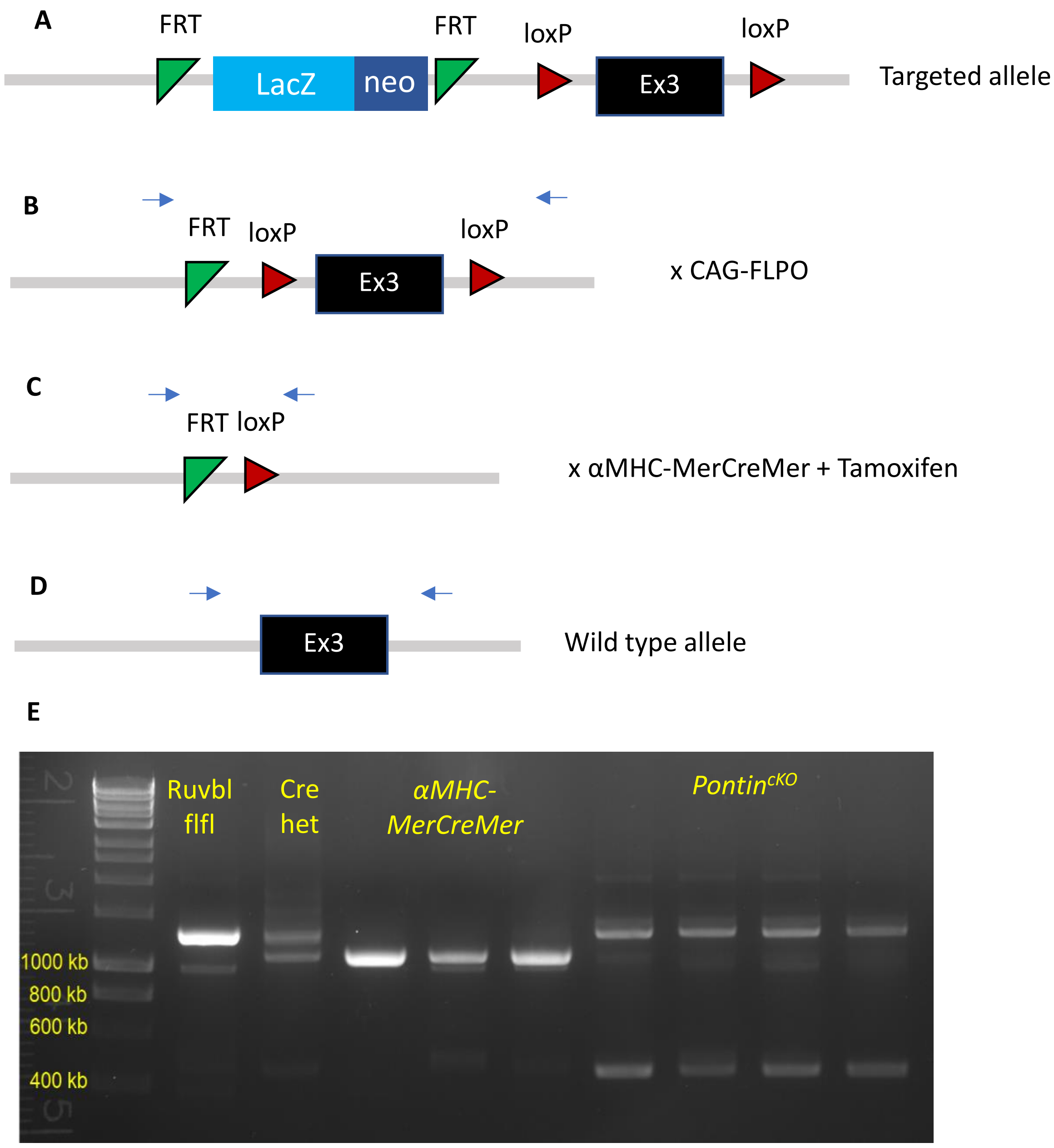

Supplementary figure 6. Strategy to generate Pontin inducible cardiomyocyte specific knockout mice (Pontin^{icKO}) . A) Schematic diagram to describe Pontin gene targeting construct. Exon 3 of the mouse *Ruvbl1* gene was targeted using construct carrying loxP sites flanking exon 3, lacZ and neo resistant markers, and flp sites flanking the lacZ/neo marker. **B**) Diagram showing the targeting construct following cross-breeding with CAG-Flpo mice to remove the lacz/neo marker resulting in Pontin^{floxed} mice. Arrows represent the primer binding sites for the genotyping. **C**) Exon 3 was deleted following cross breeding with the αMHC MerCreMer mice followed by single injection with tamoxifen (40 µg/kg BW).This will result in the generation of cardiomyocyte specific deletion of Pontin. **D**) Diagram showing the wildtype allele and the primer binding sites (arrows). **E**) Representative of PCR genotyping using primers represented by the arrows.

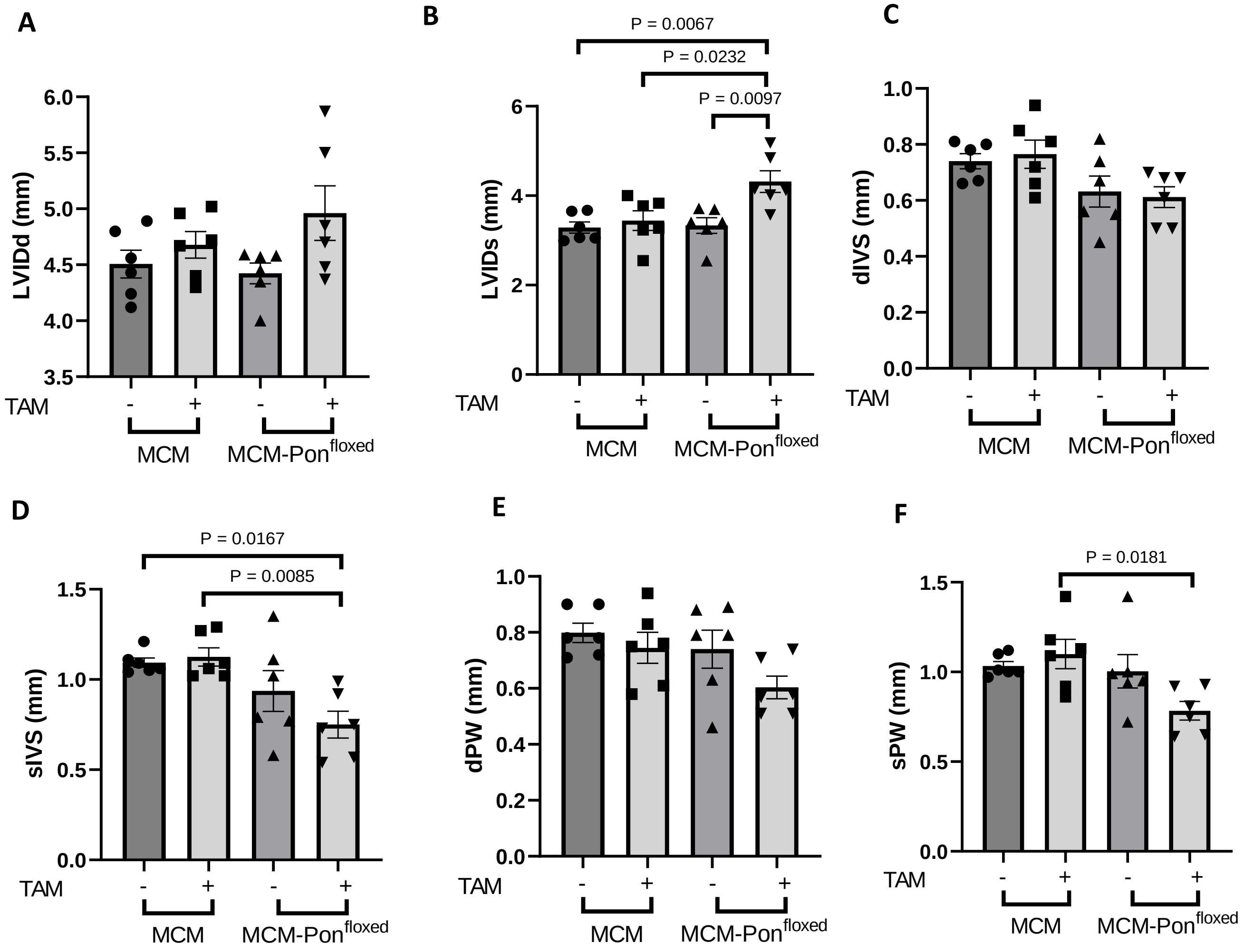

Supplementary figure 7. Echocardiography analysis to assess cardiac morphology and function of of Pontin^{icKO} mice. Cardiac morphometric data were obtained at day 24 following tamoxifen injection. **A**) Left ventricular diameter at diastole (LVID$_d$) was not different between Pontin^{icKO} mice (αMCM Pontin^{floxed} + tamoxifen) compared to other groups **B**) However, at systole there was a significant increased in LVID in Pontin^{icKO} compared to other groups. **C**) Septal wall thickness at diastole (dIVS) was not different between all groups tested. **D**) However, at systole Pontin^{icKO} mice displayed thinner wall thickness (sIVS) compared to controls. **E**) Consistently, there is no difference in posterior wall thickeness at diastole (dPW) between all group tested. **F**) Pontin icKO mice exhibited thinner posterior wall thickness at systole (sPW). (n=6 mice in each group). Data are presented as mean ± SEM.Statistical test used: one way ANOVA followed by multiple comparisons. Source data are provided as a Source Data file.

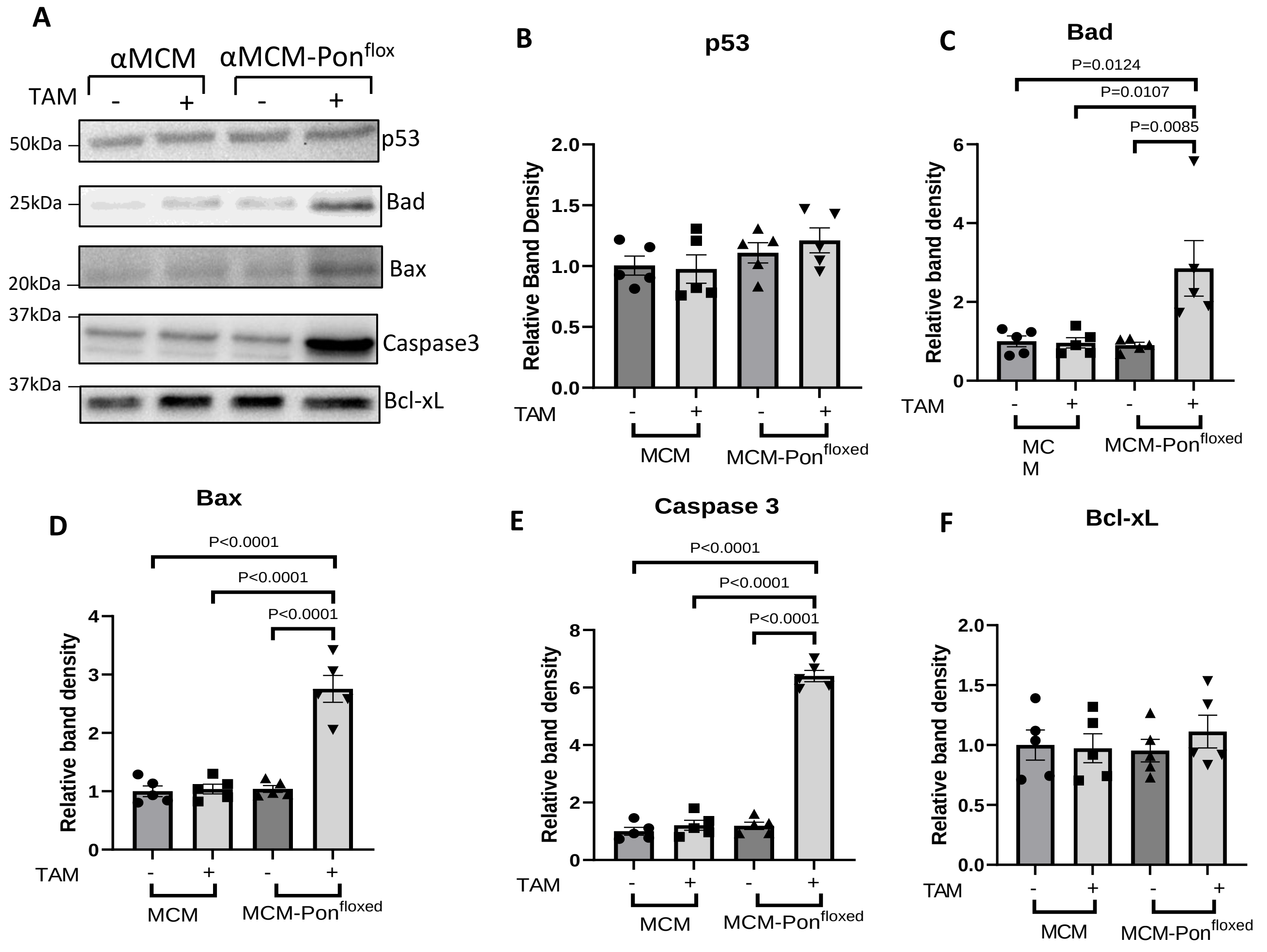

Supplementary figure 8. Expressions of apoptosis regulators in the heart of Pontin^{icKO} mice. A) Representative Western blot images showing the expression of regulators apoptosis in Pontin^{icKO} mice (αMCM Pontin^{floxed} + tamoxifen) and control groups. **B**) Quantification of band density did not show any difference of p53 expression in all groups. However, expression of **C**) Bad, **D**) Bax, and **E**) Caspase 3 were significantly increased in Pontin^{icKO} mice compared to controls. **F**) Band density measurement of Bcl-xL expression did not show any different of expression between groups. (n=5 independent experiments per group). Data are presented as mean ± SEM. Statistical test used: one way ANOVA followed by multiple comparisons. Source data are provided as a Source Data file.

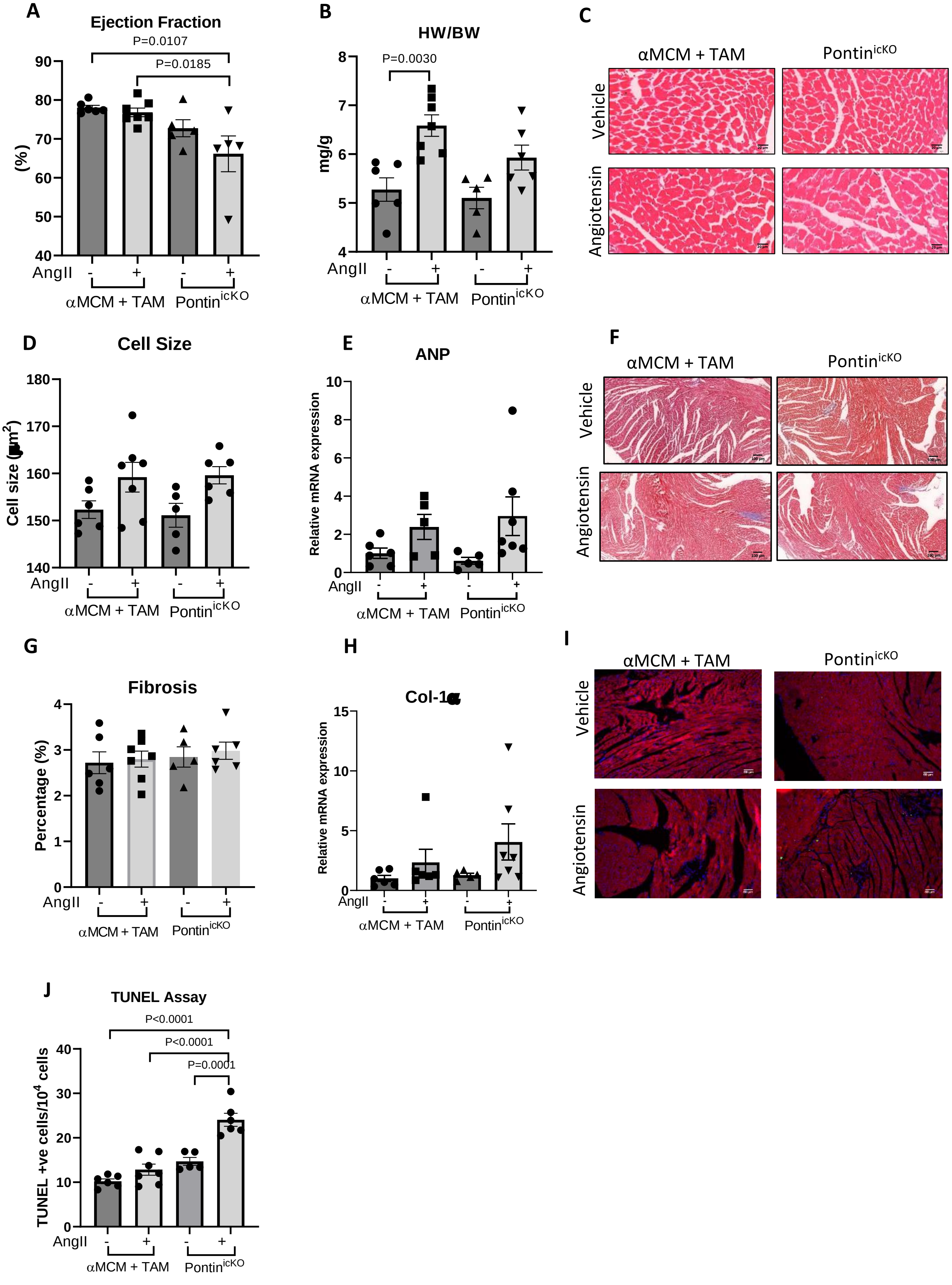

A
Ejection Fraction
P=0.0107
P=0.0185
(%)
AngII
αMCM + TAM
Pontin^icKO
B
HW/BW
P=0.0030
mg/g
C
αMCM + TAM
Pontin^icKO
Vehicle
Angiotensin
D
Cell Size
Cell size (μm²)
E
ANP
Relative mRNA expression
F
G
Fibrosis
Percentage (%)
H
Col-1α
I
J
TUNEL Assay
P<0.0001
P<0.0001
P=0.0001
TUNEL +ve cells/10⁴ cells

Supplementary figure 9. Pontin^{icKO} mice exhibited enhanced cardiomyopathy following Ang II stimulation. Pontin^{icKO} mice and controls were treated with Ang II (1.5 mg/kg BW/day). Cardiac Phenotype was observed at day 3 following mini osmotic pump implantation. **A**) Cardiac contractility as indicated by ejection fraction (EF) was significantly reduced in Pontin^{icKO} at 3 days after Ang II treatment (αMCM + Tam – vehicle, n=6; αMCM + Tam – AngII, n=7; Pontin^{icKO} vehicle, n=5; Pontin^{icKO} AngII, n=5 mice). **B**) Analysis of heart weight/body weight (HW/BW) ratio indicated a significant increase in HW/BW ratio in control group (αMCM + Tam) after Ang II treatment. However, there was no significant increase in HW/BW ratio in Pontin^{icKO} mice following Ang II stimulation (αMCM + Tam – vehicle, n=6; αMCM + Tam – AngII, n=7; Pontin^{icKO} vehicle, n=5; Pontin^{icKO} AngII, n=6 mice). **C**) Representative H&E stained histological sections (scale bars=20μm) and **D**) analysis of cardiomyocyte cell size indicated that there was no increase in cell size in all groups at day 3 post Ang II infusion (αMCM + Tam – vehicle, n=6; αMCM + Tam – AngII, n=7; Pontin^{icKO} vehicle, n=5; Pontin^{icKO} AngII, n=6 mice). **E**) qPCR analysis to detect ANP expression showed that there was no difference in ANP expression (αMCM + Tam – vehicle, n=6; αMCM + Tam – AngII, n=5; Pontin^{icKO} vehicle, n=5; Pontin^{icKO} AngII, n=7 mice). **F**) Masson's trichrome staining of cardiac sections (αMCM + Tam – vehicle, n=6; αMCM + Tam – AngII, n=7; Pontin^{icKO} vehicle, n=5; Pontin^{icKO} AngII, n=6 mice) (scale bars=100μm) , **G**) analysis of fibrotic area and **H**) expression of fibrosis marker Col1a showed that there was no increase in fibrosis level in Pontin^{icKO} mice at day 3 post Ang II treatment (αMCM + Tam – vehicle, n=6; αMCM + Tam – AngII, n=6; Pontin^{icKO} vehicle, n=5; Pontin^{icKO} AngII, n=7 mice). **I**) Representative images of TUNEL analysis (scale bars=50μm) and **J**) quantification of TUNEL-positive cardiomyocytes suggested that there was an increase in apoptosis in Pontin^{icKO} mice following Ang II treatment compared to controls (αMCM + Tam – vehicle, n=6; αMCM + Tam – AngII, n=7; Pontin^{icKO} vehicle, n=5; Pontin^{icKO} AngII, n=6 mice). Data are presented as mean ± SEM. Statistical test used: one way ANOVA followed by multiple comparisons. Source data are provided as a Source Data file.

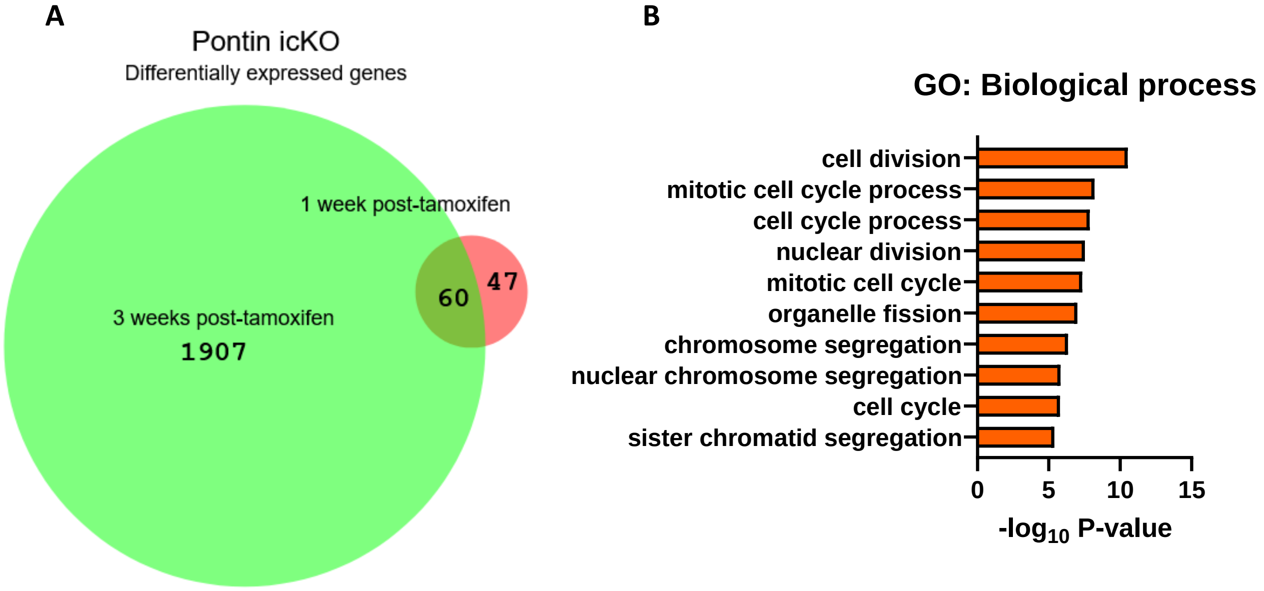

Supplementary figure 10. Analysis of genes that diffrentially expressed at both 1 week and 3 weeks after Pontin knockout in cardiomyocytes in vivo. A) Venn diagram showing the number of genes that differentially expressed at both time points following the induction of Pontin knock out. Analysis was performed using Biovenn application[1]. **B)** Functional enrichment analysis based on gene ontology database revealed that most of the genes that are differentially regulated at both time points were related to cell division and cell cycle. Analysis was performed using g:profiler web tools[2]. Source data are provided as a Source Data file.

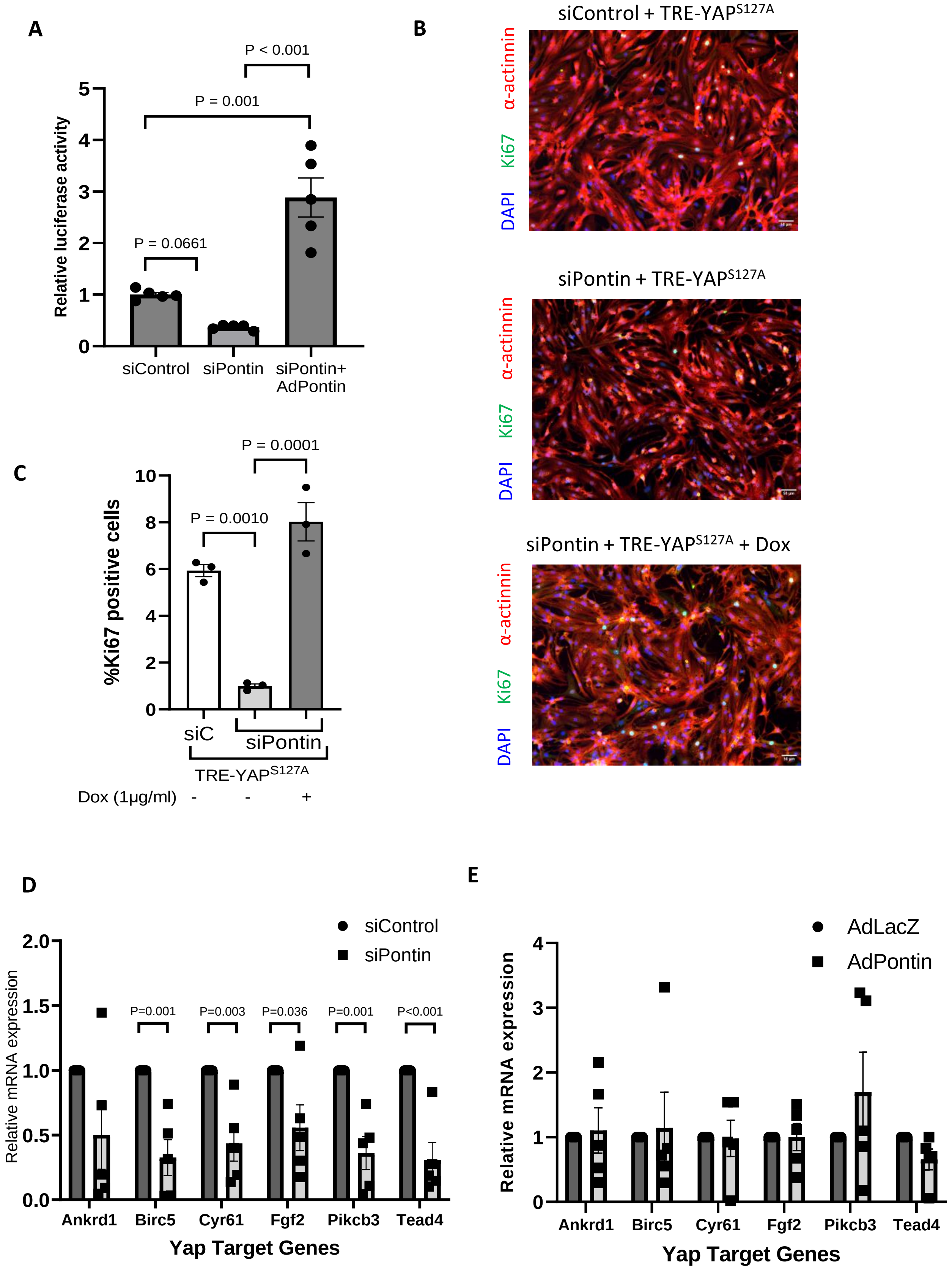
A
P < 0.001
P = 0.001
P = 0.0661
Relative luciferase activity
siControl
siPontin
siPontin+
AdPontin
B
siControl + TRE-YAP^S127A
siPontin + TRE-YAP^S127A
siPontin + TRE-YAP^S127A + Dox
α-actinnin
Ki67
DAPI
C
P = 0.0001
P = 0.0010
%Ki67 positive cells
siC
siPontin
TRE-YAP^S127A
Dox (1μg/ml) - - +
D
siControl
siPontin
P=0.001
P=0.003
P=0.036
P=0.001
P<0.001
Relative mRNA expression
Ankrd1
Birc5
Cyr61
Fgf2
Pikcb3
Tead4
Yap Target Genes
E
AdLacZ
AdPontin
Relative mRNA expression
Ankrd1
Birc5
Cyr61
Fgf2
Pikcb3
Tead4
Yap Target Genes

Supplementary figure 11. Rescue experiments by Pontin overexpression and analysis of YAP target genes in NRCM. A) Pontin gene silencing in NRCM significantly reduced YAP activity, however, re-expression of Pontin in these cells significantly enhanced YAP activity as detected by luciferase sensor (n=5 independent experiments). **B)** Representative images of Ki67 staining in NRCM and **C)** Analysis of Ki67-positive cells showing that induced overexpression of constitutively active YAP^{S127A} rescued the reduction of Ki67 positive cells following Pontin knockdown (n=3 independent experiments). **D)** Expression of known YAP target genes, such as Ankrd1, Birc5, Cyr61, Pik3cb, Fgf2 and Tead1 were detected using qRT-PCR in NRCM treated with siRNA to knockdown Pontin. Significant reduction in the expression of all of the genes above was observed in NRCM lacking Pontin (n=6 independent experiments). **E)** qRT-PCR results showing expression of YAP target genes in NRCM overexpressing Pontin. No. significant changes were observed in any gene tested (n=6 independent experiments). Data are presented as mean ± SEM. Statistical tests used: A&C, one way ANOVA followed by multiple comparisons; D&E, multiple t-test. Source data are provided as a Source Data file.

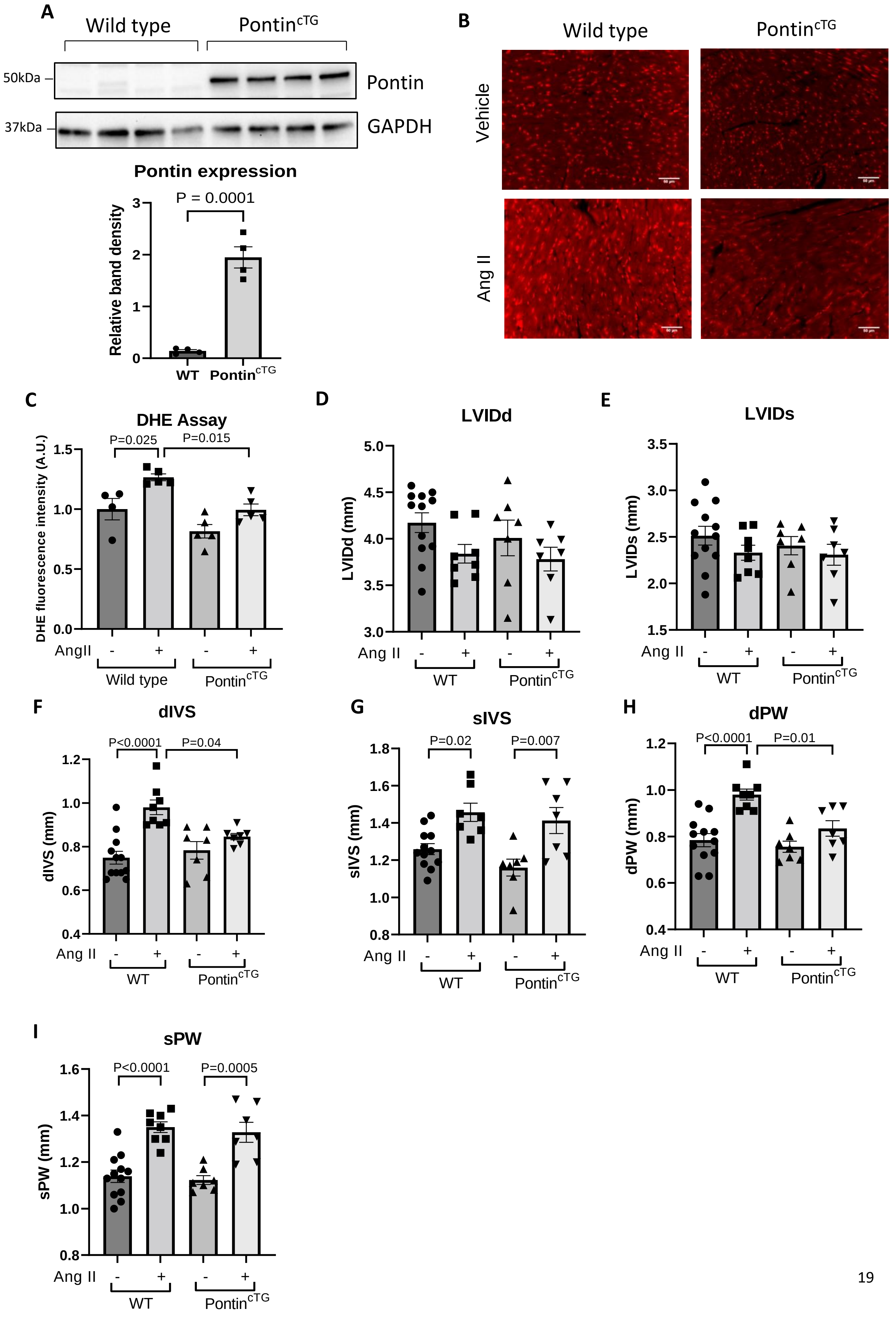

A
Wild type
Pontin cTG
50kDa
Pontin
37kDa
GAPDH
Pontin expression
P = 0.0001
Relative band density
WT
Pontin cTG
B
Wild type
Pontin cTG
Vehicle
Ang II
C
DHE Assay
P=0.025
P=0.015
DHE fluorescence intensity (A.U.)
AngII
Wild type
Pontin cTG
D
LVIDd
LVIDd (mm)
Ang II
WT
Pontin cTG
E
LVIDs
LVIDs (mm)
Ang II
WT
Pontin cTG
F
dIVS
P<0.0001
P=0.04
dIVS (mm)
Ang II
WT
Pontin cTG
G
sIVS
P=0.02
P=0.007
sIVS (mm)
Ang II
WT
Pontin cTG
H
dPW
P<0.0001
P=0.01
dPW (mm)
Ang II
WT
Pontin cTG
I
sPW
P<0.0001
P=0.0005
sPW (mm)
Ang II
WT
Pontin cTG

Supplementary figure 12. Transgenic overexpression of Pontin protected against Ang II induced pathological changes in the heart. **A)** Western blot analysis using anti-Pontin antibody and quantification of band density showing a signicicant overexpression of Pontin in the hearts of Pontin^{cTG} mice (n=4 mice in each group). **B**) Representative images of heart tissue sections stained with DHE to examine the level of oxidative stress. **C**) Quantification of fluorescence signal revealed that Pontin^{cTG} mice displayed lower level of oxidative stress (WT vehicle, n=4; WT Ang II, n=5, Pontin^{cTG} vehicle, n=5, Pontin^{cTG} Ang II, n=5 mice). **D**) Echocardiography analysis of Pontin^{cTG} mice and controls at 2 weeks after Ang II stimulation (1.5 mg/kBW/day). Measurement of **D**) LVIDd, **E**) LVIDs, **F**) dIVS, **G**) sIVS, **H**) dPW, **I**) sPW showed that Pontin^{cTG} mice displayed less hypertrophy as indicated by thinner dIVS and dPW compared to WT controls (WT vehicle, n=11; WT Ang II, n=8, Pontin^{cTG} vehicle, n=7, Pontin^{cTG} Ang II, n=7 mice). Data are presented as mean ± SEM. Statistical tests used: B, Student's t-test; C-I, one way ANOVA followed by multiple comparisons. Source data are provided as a Source Data file.

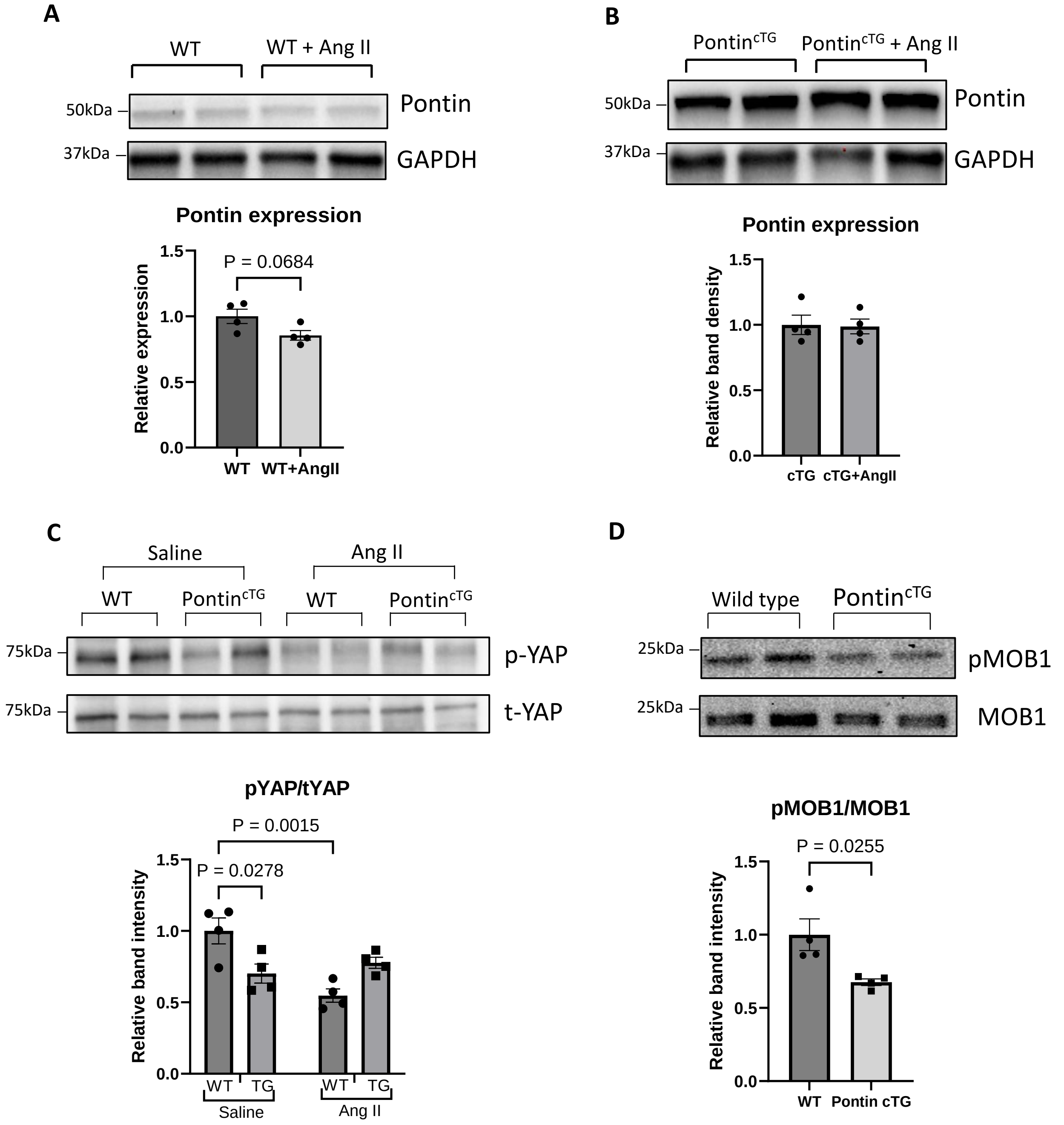

Supplementary figure 13. Transgenic overexpression of Pontin in mouse hearts modulates Hippo/YAP signalling pathway. A) There was a trend of reduced **c**ardiac expression of Pontin in WT mice in response to Ang-II treatment (1.5 mg.kg BW/day for two weeks)(n=4 in each group). **B)** Expression of Pontin in Pontin^{cTG} mice did not change following treatment with Ang-II (n=4 mice in each group). **C**) Western blot analysis suggested that the level of phospho/total-YAP was significantly reduced in Pontin^{cTG} mice vs WT in control condition (saline). Phospho/total-YAP level was reduced in WT mice in response to Ang-II treatment, indicating enhancement of YAP activity. However, there was no change in phospho/total-YAP level in Pontin^{cTG} mice following Ang-II stimulation (n=4 mice in each group). **D**) The level of phospho/total MOB1 was reduced in Pontin^{cTG} hearts. Data are presented as mean ± SEM. Statistical tests used: A,B,D, Student's t-test; C, one way ANOVA followed by multiple comparisons. Source data are provided as a Source Data file.

SUPPLEMENTARY REFERENCES

1. Hulsen T, de Vlieg J, Alkema W. BioVenn - a web application for the comparison and visualization of biological lists using area-proportional Venn diagrams. *BMC Genomics* **9**, 488 (2008).

2. Kolberg L, Raudvere U, Kuzmin I, Adler P, Vilo J, Peterson H. g:Profiler-interoperable web service for functional enrichment analysis and gene identifier mapping (2023 update). *Nucleic Acids Res* **51**, W207-W212 (2023).
