# Supplementary material for: Expression of foetal gene Pontin is essential in protecting heart against pathological remodelling and cardiomyopathy
Source: Nat Commun. 2025 Feb 14;16:1650. doi: 10.1038/s41467-025-56531-4 (PMC11829043; doi:10.1038/s41467-025-56531-4)
Supplement: Supplementary file 3 — Reporting Summary [file 41467_2025_56531_MOESM3_ESM.pdf]

nature portfolio

Corresponding author(s): Delvac Oceandy

Last updated by author(s): Dec 12, 2024

Reporting Summary

Nature Portfolio wishes to improve the reproducibility of the work that we publish. This form provides structure for consistency and transparency in reporting. For further information on Nature Portfolio policies, see our Editorial Policies and the Editorial Policy Checklist.

Statistics

For all statistical analyses, confirm that the following items are present in the figure legend, table legend, main text, or Methods section.

n/a	Confirmed	
☐	☒	The exact sample size (*n*) for each experimental group/condition, given as a discrete number and unit of measurement
☐	☒	A statement on whether measurements were taken from distinct samples or whether the same sample was measured repeatedly
☐	☒	The statistical test(s) used AND whether they are one- or two-sided<br>*Only common tests should be described solely by name; describe more complex techniques in the Methods section.*
☒	☐	A description of all covariates tested
☐	☒	A description of any assumptions or corrections, such as tests of normality and adjustment for multiple comparisons
☐	☒	A full description of the statistical parameters including central tendency (e.g. means) or other basic estimates (e.g. regression coefficient) AND variation (e.g. standard deviation) or associated estimates of uncertainty (e.g. confidence intervals)
☐	☒	For null hypothesis testing, the test statistic (e.g. *F*, *t*, *r*) with confidence intervals, effect sizes, degrees of freedom and *P* value noted<br>*Give P values as exact values whenever suitable.*
☒	☐	For Bayesian analysis, information on the choice of priors and Markov chain Monte Carlo settings
☒	☐	For hierarchical and complex designs, identification of the appropriate level for tests and full reporting of outcomes
☒	☐	Estimates of effect sizes (e.g. Cohen's *d*, Pearson's *r*), indicating how they were calculated

Our web collection on statistics for biologists contains articles on many of the points above.

Software and code

Policy information about availability of computer code

Data collection	N/A
Data analysis	GraphPad Prism Software ver 10.2.2. (GraphPad Software, LLC)

For manuscripts utilizing custom algorithms or software that are central to the research but not yet described in published literature, software must be made available to editors and reviewers. We strongly encourage code deposition in a community repository (e.g. GitHub). See the Nature Portfolio guidelines for submitting code & software for further information.

Data

Policy information about availability of data

All manuscripts must include a data availability statement. This statement should provide the following information, where applicable:

- Accession codes, unique identifiers, or web links for publicly available datasets
- A description of any restrictions on data availability
- For clinical datasets or third party data, please ensure that the statement adheres to our policy

All of the data from this study are presented in the 'Results' section and Supplemental Materials of this paper. The data generated in this study are provided in Source Data file. RNA seq data for PontinicKO experiment have been deposited in the ArrayExpress database (http://www.ebi.ac.uk/arrayexpress) under accession number E-MTAB-14659. RNA seq data for PontincTG experiment have been deposited in NCBI's Gene Expression Omnibus under accession number GSE282958.

Research involving human participants, their data, or biological material

Policy information about studies with human participants or human data. See also policy information about sex, gender (identity/presentation), and sexual orientation and race, ethnicity and racism.

Reporting on sex and gender	Human heart tissues were obtained from Asterand. Information on gender is available in suplementary table S1.
Reporting on race, ethnicity, or other socially relevant groupings	No information on race, ethnicity and social groupings are available
Population characteristics	Information on clinical diagnosis are available in Supplementary Table 1
Recruitment	Tissues were obtained from Asterand.
Ethics oversight	Ethical clearance is obtained by Asterand.

Note that full information on the approval of the study protocol must also be provided in the manuscript.

Field-specific reporting

Please select the one below that is the best fit for your research. If you are not sure, read the appropriate sections before making your selection.

☒ Life sciences ☐ Behavioural & social sciences ☐ Ecological, evolutionary & environmental sciences

For a reference copy of the document with all sections, see nature.com/documents/nr-reporting-summary-flat.pdf

Life sciences study design

All studies must disclose on these points even when the disclosure is negative.

Sample size	For the in vivo experiments, sample size was determined based on Pilot data using Pontin transgenic mice. Sample size calculations at a power of 80% with alpha of 0.05 (two sided t-test) suggest that a minimum of n=5 mice per group are required. This sample size calculation was used for the analysis of cardiac phenotype of Pontin knockout and transgenic mice.
Data exclusions	No data were excluded
Replication	All the findings were replicated as indicated in the figure legends.
Randomization	For in vivo experiments, mice were randomly selected to be included in each experimental group, e.g. treatment with AngII to induce hypertrophy or with tamoxifen to delete Pontin). For in vitro experiments, cells in each cultured TC well were randomly selected for treatment (overexpression or gene silencing).
Blinding	The investigators were blinded in analysing the in vivo data, such as echocardiography. For in vitro experiments, there was no blinding process since the same researcher performed both treatment and phenotype analysis.

Reporting for specific materials, systems and methods

We require information from authors about some types of materials, experimental systems and methods used in many studies. Here, indicate whether each material, system or method listed is relevant to your study. If you are not sure if a list item applies to your research, read the appropriate section before selecting a response.

Materials & experimental systems

n/a	Involved in the study	
☐	☒	Antibodies
☐	☒	Eukaryotic cell lines
☒	☐	Palaeontology and archaeology
☐	☒	Animals and other organisms
☒	☐	Clinical data
☒	☐	Dual use research of concern
☒	☐	Plants

Methods

n/a	Involved in the study	
☒	☐	ChIP-seq
☒	☐	Flow cytometry
☒	☐	MRI-based neuroimaging

Antibodies

Antibodies used	List of antibodies and dilutions are described in Supplementary Table 2
Validation	Information on validation are available in the manufacturer's website.

Eukaryotic cell lines

Policy information about cell lines and Sex and Gender in Research

Cell line source(s)	H9c2 cells were obtained from ATCC
Authentication	We did not perform authentication procedure
Mycoplasma contamination	Cells were tested negative for mycoplasma contamination
Commonly misidentified lines (See ICLAC register)	N/A

Animals and other research organisms

Policy information about studies involving animals; ARRIVE guidelines recommended for reporting animal research, and Sex and Gender in Research

Laboratory animals	Mouse (C57Bl/6 background) and rat (Sprague Dawley)
Wild animals	N/A
Reporting on sex	Cardiac remodelling process is affected by sex. We performed experiments in male mice as described in methods section.
Field-collected samples	N/A
Ethics oversight	Animal maintenance and experiments were conducted in accordance with the United Kingdom Animals (Scientific Procedures) Act and approved by the University of Manchester Ethics Committee.

Note that full information on the approval of the study protocol must also be provided in the manuscript.

Plants

Seed stocks	N/A
Novel plant genotypes	N/A
Authentication	N/A
